# Modeling and Characterizing the Growth of the Texas–New Mexico Measles Outbreak of 2025

**DOI:** 10.3390/epidemiologia6040060

**Published:** 2025-10-03

**Authors:** Gilberto González-Parra, Annika Vestrand, Remy Mujynya

**Affiliations:** Department of Mathematics, New Mexico Tech, Socorro, NM 87801, USAremy.mujynya@student.nmt.edu (R.M.)

**Keywords:** measles outbreak, mathematical modeling, growth rate, effective reproduction number

## Abstract

Background: In late January 2025, a measles outbreak began in Gaines County, Texas, USA, and the outbreak extended to New Mexico. We used a variety of mathematical models to estimate the growth rate of the Texas–New Mexico measles outbreak of 2025. Methods: We used both empirical and mechanistic models based on differential equations to make the estimations that allow us to characterize this measles outbreak. Regarding empirical models, we used the exponential growth model to compute and estimate the growth rate, basic reproduction number, $R_{0}$, and effective reproduction number $R_{t}$. With regard to mechanistic models, we use the SIR and SEIR models to estimate the growth rate, basic reproduction number $R_{0}$, and effective reproduction number $R_{t}$. We used new weekly measles cases and also cumulative cases. Results: Using the exponential growth model, we estimated a basic reproduction number between 32 and 40. For the classical SIR model, we estimated that the basic reproduction number is approximately 30. Conclusion: We found that the current Texas–New Mexico measles outbreak of 2025 has a slightly higher growth rate and effective reproduction number $R_{t}$ compared to several previous measles outbreaks around the world.

## 1. Introduction

In late January 2025, a measles outbreak started in Gaines County, Texas. In particular, the outbreak began in a Mennonite community where there are low levels of childhood immunization. By 23 March, Texas had reported cases in 14 counties. The measles outbreak also expanded to New Mexico in some counties adjacent to West Texas [1,2]. In particular, the first cases in New Mexico were in Lea County, which borders the Gaines County hot spot in West Texas. There were a particularly high number of cases in Texas and New Mexico during this measles outbreak. People were hospitalized, and some died. One death was a six-year-old girl who was not vaccinated [3,4]. The outbreak also spread to Oklahoma. Some officials suspect that the true number of cases is higher, since there are generally one to two deaths per 1000 cases. The underreporting situation is also common with respect to many other infectious diseases such as influenza and COVID-19 [5]. Undereported cases often make it more difficult to stop outbreaks. The annual number of measles cases in the US is currently the highest in 33 years and there is an ongoing measles outbreak. The Centers for Disease Control and Prevention (CDC) reports 1267 cases of measles and, as of 4 July, Johns Hopkins University’s Center for Outbreak Response Innovation had reported 1277 cases of measles [6,7].

The Texas–New Mexico outbreak occuredin the western part of Texas and in the eastern part of New Mexico. Around the world, there have been other outbreaks, as well as in other states of the USA. The Center for Disease Control and Prevention (CDC) has reported more cases during 2025 than during the whole of 2024. An important aspect of the Texas–New Mexico measles outbreak is that at one point in the outbreak of 309 cases, 307 were unvaccinated or had an unknown vaccination status [8].

Measles is highly contagious; therefore, more cases are expected during the Texas–New Mexico 2025 measles outbreak. This outbreak may be due to low vaccination coverage, insufficient administered doses of the measles (MMR) vaccine, groups of intentionally under-vaccinated children, and/or imported measles from global travel [9].

One way to measure the force of outbreaks is by estimating the growth rate, the basic reproduction number, or the effective reproduction number [10,11,12,13]. The exponential growth rate in some way measures the speed of spread of an infectious disease [13,14]. The basic reproduction number $R_{0}$ has been estimated for many epidemics in different regions of the world [15,16,17]. There are advantages to each of the previous metrics in measuring the force of epidemics. For example, the growth rate *r* measures the speed of epidemic growth, which provides information on the time scale of the spread of the disease [14]. On the other hand, the basic reproduction number $R_{0}$ does not have an associated time scale. Therefore, epidemics with the same basic reproduction number can occur during very different time periods [14]. There are many obvious reasons why the time scale is relevant from different points of view. For example, the growth rate in the classical exponential model changes over time. Furthermore, the growth rate is independent of the generation interval distribution. Similarly, the effective reproduction number changes over time [15].

The basic reproduction number $R_{0}$ represents the number of secondary infections caused by a single infected person in a fully susceptible population [15]. A higher basic reproduction number means that the virus can spread to many people and poses a high risk to public health. Thus, estimating the basic reproduction number is very useful in measuring the risk of epidemics. Another aspect related to $R_{0}$ is that an increase in $R_{0}$ by 0.4 can enhance the transmissibility of the disease by 50–70% with a four-fold increase in viral load [18,19]. With regard to the time-varying effective reproduction number $R_{t}$, it measures the number of new infections caused on average by a single infection at a particular time. Thus, the lower the number of susceptible people, the lower this number, since less people can become infected [20,21,22,23,24]. The effective reproduction number $R_{t}$ is also useful for outbreaks in which a certain percentage of the population is immune to the disease due to vaccination or previous exposure. Thus, for the current measles outbreak it is relevant, since a large proportion of the population is immune to measles due to vaccination [25]. In this work, we estimate the growth rate, the basic reproduction number, and the effective reproduction number. Some equations provide the relationships between these quantities under some underlying assumptions [14,25,26,27,28]. For example, using the exponential growth rate model, it is possible to estimate the basic reproduction number $R_{0}$ using the value of the first generation interval and the growth rate [17,29]. In addition, the effective reproduction number $R_{t}$, can be computed from the growth rate *r* and the distribution of the generation-interval [30].

Previous studies related to the dynamics of measles have been presented [31,32,33]. Estimates of the exponential growth rate, the basic reproduction number, and the effective reproduction number are available in different works [25,34]. In [34], a with-in-host model for measles was developed to investigate a case study.

With regard to the incubation and infectious period of measles, there are a variety of results in the scientific literature [25,35]. For example, in [35], it was mentioned that the infectious period lasts approximately five days. However, an unusually long incubation period of at least 23 days has been reported [35]. In [36], it was mentioned that the period of infectivity starts from the last two days of incubation until four days after the rash appears. In [37], a model was used to study measles and one week was assumed for the infectious period. The basic reproduction number was estimated as 12.8 and the basic reproduction with immunization was estimated as 2.85. In [32], a stochastic model was used to determine whether or when it is worth using outbreak-response vaccination programs in schools. The authors assumed an incubation period of 9.97 days and an infectious period of 8.7 days. In [38], the authors used two such datasets and found that the mean incubation period lies in the range of 11–12 days. However, in [39], an incubation period of 3.5 days was also used in a mathematical modeling study regarding measles.

Measles is highly contagious, and therefore, it is very likely that unimmunized individuals will become infected when exposed to an infected person. It is important to mention that there are a variety of strains of measles. However, recovery from infection from one strain provides life-immunity against other strains [40,41]. Thus, for measles, the combination of life-immunity and highly contagious translates into a predominantly childhood disease dependent on births [40].

Previous works have proposed a variety of models to study measles dynamics using different points of view [25,32,40]. Some works have used the SIR and SEIR models [33,42,43]. The use of these models is suitable for measles as it is a disease for which acquired immunity is permanent [44]. In [33], it is mentioned that for the SIR model, the infectious period can be assumed to be 13 days, whereas for the SEIR model, the exposed stage lasts 8 days with an infectious period of 5 days. In [43], the authors used an exponentially distributed infectious period of 7 days and an exponentially distributed latent period between 2 and 11 days. The authors also assumed that the generation time for measles is approximately 14 days. In [45], an effective reproduction number between 12.5 and 18 was reported. In [31], a systematic review of works that have estimated the basic reproduction number $R_{0}$ of measles is presented. The review covered works that made estimations pre-vaccine era and post-vaccine era. Thus, estimates of the effective reproduction number $R_{t}$ are also presented. With regard to the former, a basic reproduction number $R_{0}$ for measles has been reported to be up to 770 and a minimum value of 1.43. For the post-vaccine era, a maximum value of 32.1 and a minimum value of 4.6. All these results show that the usual range of 12–18 is not always reliable. Thus, in [31], the authors highlight the importance of estimating the basic reproduction number for different regions around the world using local data. In this way, we can provide more robust data and help to understand the dynamics of measles.

Based on these previous ideas, in this work, we aim to provide estimates of the growth rate, the basic reproduction number, and the effective reproduction number for the early phase of the Texas–New Mexico 2025 outbreak. During the early phase, we expect that changes in human behavior or non-pharmaceutical interventions have not fully occurred. We rely on methods that have been used in other works [13,14,25,26,27,28]. Oftentimes, these methods have underlying assumptions that may not be suitable for certain scenarios and could be one of the reasons there is a wide range of reported values of the basic reproduction number $R_{0}$ of measles [31].

## 2. Materials and Methods

In this section, we present both empirical and mechanistic models based on differential equations to estimate the growth rate of the Texas and New Mexico measles outbreak of 2025. With regard to empirical models, we use the exponential growth model to compute and estimate the growth rate, basic reproduction number, $R_{0}$, and effective reproduction number $R_{t}$. With regard to mechanistic models, we use the SIR and SEIR models to estimate the growth rate, basic reproduction number, $R_{0}$, and effective reproduction number $R_{t}$. All these aforementioned mathematical models are fitted to to CDC’s weekly measles datasets for new and cumulative cases [6,46].

### 2.1. Exponential Growth Model

The exponential growth model is suitable for estimating the growth rate in the early phase of an epidemic in which the cases are increasing [11,12,13,17]. However, the growth rate can vary during the growth phase of the epidemic and depends on the window used to estimate the growth rate [14,26]. However, it is not suitable when the cases are in the dropping or in the decay phase. One main drawback of the exponential growth model is that it does not offer an explanation for the underlying biological process for the measles outbreaks. Despite this limitation, the model has been used to estimate the growth rate when epidemic outbreaks start [11,12,17,47]. The exponential growth model can be written as follows [12]:

(1) $$I^{\prime }\left(t\right)=rI\left(t\right),$$

where *r* is the exponential growth rate and *t* is time. The solution to this ODE is given by

(2) $$I\left(t\right)=I_{0}\;e^{rt},$$

where $I_{0}$ is the initial number of cases at t=0. This number of cases often has uncertainty and there are a variety of approaches to deal with this [5,48]. Due to the clear symptoms of measles, this uncertainty appears to be reduced for the Texas–New Mexico measles outbreak.

The exponential growth rate itself can be used to compare measles outbreaks in different regions. Moreover, if the recovery rate for measles is known and the entire population is susceptible, then the recovery rate can be used to compute or estimate the transmission rate of measles using β=r+γ, where β is the transmission rate and γ is the infectious period [14,15]. This equation also allows the calculation of the basic reproduction number $R_{0}$ [14,15]. However, for the Texas–New Mexico measles outbreak, a large proportion of the population is immune to measles and therefore a small proportion is susceptible. Therefore, we need to rely on another approach.

The generation interval method has been used extensively to compute the effective reproduction number $R_{t}$ [25,49]. The exponential growth *r* is related to the speed of infection at the population level, and the generation interval is also related to the speed but at the individual level [25]. Generation interval distributions might be approximated from contact tracing when possible. If not, there are many distributions that can be used, but many other works have chosen a gamma distribution because of its versatility. In [50], the Weibull and gamma distributions provided similar results for the mean of the generation interval (COVID-19 data), but the Weibull distribution provided lower values for the standard deviation. The gamma distribution generalizes the result obtained from simple SIR models and the SEIR model approximately (when the latent period and infectious period are similar) [25,51]. In addition, the gamma distribution has two parameters with biological meaning (shape and scale), which allow us to approximately resemble many other distributions. In [25], a very interesting work is presented in which the relationships between the speed of the epidemic growth and the strength of the epidemic are revealed. Using the exact speed–strength relationship presented in [52], the authors used the particular gamma distribution to obtain the following gamma-approximated speed–strength relationship [25]:

(3) $$R_{t}\approx {\left(1+\kappa r\bar{G}\right)}^{1/\kappa },$$

where $\bar{G}$ is the mean of the gamma distribution, κ is the squared coefficient of variation (κ=1/a), and *r* is the exponential growth (or rate of spread of the disease). The gamma distribution can be given in terms of the shape parameter *a* and the scale parameter θ. Then, the mean of the gamma distribution is given by $\bar{G}=a\theta$ and the variance is given by $a\theta^{2}$ [53]. Thus, based on Equation (3), if we estimate the growth rate *r* and assume a particular gamma distribution (with parameters *a* and θ), one can approximate the value of $R_{t}$.

In [25], a new parameter ρ was introduced to express the effective reproduction number $R_{t}$. In particular, using $\rho =r\bar{G}$, one obtains

(4) $$R_{t}\approx {\left(1+\kappa \rho \right)}^{1/\kappa }.$$

Note that if κ=0, the generation interval is fixed. On the other hand, if κ increases, then some new cases are generated before and some after the mean generation time. Thus, those early cases have a greater effect and we would have $R_{t}<exp\left(\rho \right)$ since people would not need to generate many infections to maintain the growth rate [25]. In summary, a shorter generation interval results in a faster epidemic with a higher growth rate *r*. If *r* is fixed and known, then shorter disease generations would cause a lower value of $R_{t}$ (more individual generations to produce the same spread) [25]. Finally, we can also use the expression $R_{0}=\beta /\gamma$ to obtain the basic reproduction number and then use $R_{t}=R_{0}\;S\left(t\right)/N$ to estimate the effective reproduction number. In Section 3, we will use the different expressions presented here to estimate the basic reproduction number $R_{0}$.

### 2.2. SIR Model

The SIR model is one of the simplest and most well-known epidemiological models [15]. This model is based on a system of differential equations. There are variations in the model that are sometimes recognized as SIR-type models. The model divides the entire population into three classes: *S*, susceptible individuals, *I*, infectious individuals, and *R*, recovered individuals. Thus, the mechanistic SIR model without demographic factors is given as follows:

(5) $$\begin{array}{cc} S^{\prime }\left(t\right) & =-\beta S(t)\;I(t)/N,\\ I^{\prime }\left(t\right) & =\beta S(t)\;I(t)/N-\gamma I(t),\\ R^{\prime }\left(t\right) & =\gamma \;I(t). \end{array}$$

This model can be extended by including an additional equation $C^{\prime }\left(t\right)=\beta S\left(t\right)\;I\left(t\right)/N$, where C(t) is an auxiliary variable that represents the cumulative cases at time *t* [12,15]. We can calibrate the SIR model using epidemiological data. Thus, one can obtain an estimate of the parameter β if the value of the infectious phase γ (tim$e^{-1}$) is known [43]. In addition, we can estimate the basic reproduction number $R_{0}$ and the effective reproduction number $R_{t}$ [15], in particular, using $R_{0}=\beta /\gamma$ and $R_{t}=R_{0}\;S\left(0\right)/N$. Numerically solving the SIR model needs the initial conditions (at t=0) for the susceptible S(0), infected I(0), and recovered R(0) populations.

The SIR model (5) assumes an exponential transition between the *I* and *R* classes. However, an SIR or SEIR model with gamma-distributed transitions has been argued to be more realistic, but the model is larger and has additional parameters [54,55].

### 2.3. SEIR Model

In this subsection, we briefly present the classcial mechanistic SEIR model without demographics (due to the short time span of the outbreak) [15,56,57]. This model includes a latent phase in which the person has the virus but cannot spread the virus to others. An important feature in measles transmission is its incubation period [35,38,51].

The SEIR model is also based on a system of differential equations. The model divides the population into four classes: *S*, susceptible individuals, *E*, latent individuals, *I*, infectious individuals, and *R*, recovered individuals. The SEIR model without demographics can be written as follows [15,58]:

(6) $$\begin{array}{cc} S^{\prime }\left(t\right) & =-\beta \frac{S(t)I(t)}{N}\\ E^{\prime }\left(t\right) & =\beta \frac{S(t)I(t)}{N}-\sigma E\left(t\right)\\ I^{\prime }\left(t\right) & =\sigma E(t)-\gamma I(t)\\ R^{\prime }\left(t\right) & =\gamma \;I(t). \end{array}$$

Again, we can extend the model by including an additional equation $C^{\prime }\left(t\right)=\sigma E\left(t\right)$, which allows us to compute the cumulative cases at time *t* [12,15]. Thus, we can calibrate the SEIR model using new cases or cumulative cases. Thus, an estimate of the parameter β can be obtained if the value of the infectious phase γ is known. In addition, we can estimate the basic reproduction number $R_{0}$ and the effective reproduction number $R_{t}$ [15], in particular, using $R_{0}=\beta /\gamma$ and $R_{t}=R_{0}\;S\left(0\right)/N$. Numerically solving the SEIR model requires the initial conditions (at t=0) for the susceptible S(0), latent E(0), infected I(0), and recovered R(0) populations.

Note that the expression for $R_{0}$ is the same as in the SIR model when demographics are not taken into account. However, when fitting the SEIR model, the latent period 1/σ (time) affects the estimation of the transmission parameter β.

## 3. Results

This section is devoted to the results of using the different mathematical models to estimate the initial exponential growth rate, the basic reproduction number $R_{0}$, and the effective reproduction number $R_{t}$ for the Texas–New Mexico measles outbreak of 2025. We will start with the exponential growth model (1) and then continue with the mechanistic SIR and SEIR mathematical models. All these aforementioned mathematical models are fitted to to CDC’s weekly measles datasets for new and cumulative cases [6,46].

### 3.1. Exponential Growth Model Results

We estimate the exponential growth rate and the basic reproduction number $R_{0}$ by fitting the exponential growth model to CDC’s weekly measles dataset for cases [6,46]. We use MATLAB R2024a *fminsearch* built-in function to minimize the SSR, but we can rely on other software or even functions. First, we minimize the Sum of Squared Residuals (SSRs) using the following objective function:

(7) $$\min_{r}SSR=\min_{r}\sum_{j}{\left({\hat{I}}_{j}-I_{0}\;e^{r\;t_{j}}\right)}^{2},$$

where $t_{j}$ denotes time at week *j*, ${\hat{I}}_{j}$ denotes the real data for new cases of week *j*, *r* is the exponential growth rate, and $I_{0}$ represents the initial number of infected cases. The exponential growth model is calibrated using only the time period when the measles cases were increasing. Note that it may be possible that the initial number of reported cases of measles differs from $I_{0}$. However, if the proportion of unreported stays constant, that would not affect the computation of the growth rate of the outbreak. We also fitted the exponential growth model to cumulative cases to provide a comparison and a broader analysis, although in the cumulative fits, the later weeks dominate SSR.

**Figure 1:**
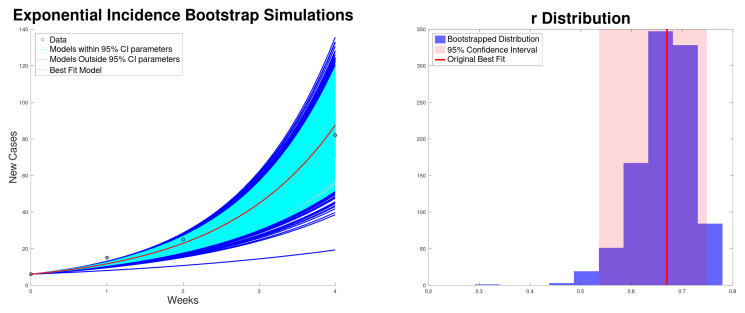
Best fit of the new cases, along with the 95% confidence interval (bootstrapping) of the exponential growth model (2) of the Texas–New Mexico measles 2025 outbreak (**left**). Bootstrapping distribution of the growth rate *r* with the 95% confidence interval (**right**).

**Figure 2:**
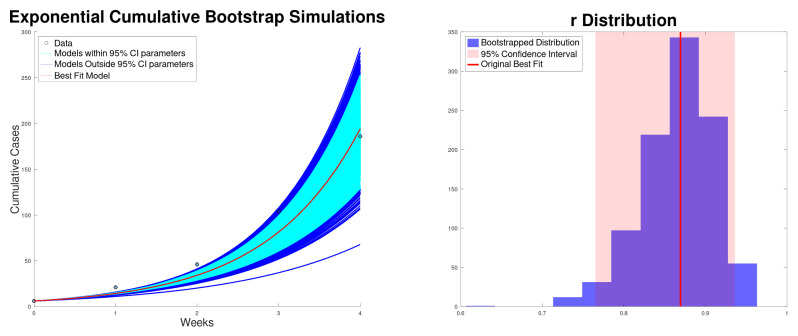
Best fit of the cumulative cases, along with the 95% confidence interval (bootstrapping) of the exponential growth model (2) of the Texas–New Mexico measles 2025 outbreak (**left**). Bootstrapping distribution of the growth rate *r* with the 95% confidence interval (**right**).

Figure 1 shows the best fit to the new cases, along with the 95% confidence interval of the exponential growth model (2) of the Texas–New Mexico measles 2025 outbreak. The confidence intervals are computed using bootstrapping [59,60]. In addition, it shows the histogram for the exponential growth rate *r*. On the other hand, Figure 2 shows the best fit to cumulative cases, along with the 95% confidence interval and the distribution of the exponential growth rate *r*. As expected, the exponential growth rate *r* obtained when fitting the cumulative data is larger than the one obtained when fitting new cases [14,26]. This is due to the fact that in the exponential model the recovered cases are not taken into account and therefore *r* can be inflated.

Table 1 shows the results of the calibrations of the exponential growth model (2) for the Texas–New Mexico measles 2025 outbreak. In particular, it shows the estimated growth rate, the estimated effective reproduction number $R_{t}$, and the basic reproduction number $R_{0}$, along with their 95% confidence intervals. We used the bootstrapping method to obtain these confidence intervals [59,60]. In addition, the SSR value is shown for the reproducibility of the results as recommended in scientific works [61]. The effective reproduction number $R_{t}$ is computed by Equation (3), but Equation (4) can also be used. Equation (3) is equivalent to

(8) $$R_{t}=\left(1+\left(1/a\right)\;\bar{G}\;r\right){)}^{a}.$$

where $\bar{G}$ is the mean of the gamma distribution. Now, using a mean of 11 days and variance of 5 day$s^{2}$ for the gamma distribution, we can obtain the estimated effective reproduction number $R_{t}$ since we estimate the value of the growth rate *r*. However, if we use a mean of 14 days, the basic reproduction number increases to 57.9. On the other hand, if we reduce the mean of the gamma distribution $\bar{G}$, the basic reproduction number (using Equations (10) and (8)) decreases below 31 and 40 for the incidence and cumulative fits. Thus, despite some uncertainty in the parameters, we still obtain results that agree well with previous results [31,54].

It is important to note that the basic reproduction number $R_{0}$ cannot be calculated directly, since most people have measles immunity in the Texas–New Mexico 2025 outbreak. This is mainly due to vaccination programs against measles [4,31,62]. Table 1 shows the results of the calibrations for both incidence and cumulative data. Alternatively, via Equation (8), we can calculate the effective reproduction number $R_{t}$ using the following generation interval equation [26,29]:

(9) $$R_{t}=\frac{I_{i}}{\sum_{j=0}^{i}\left(\rho_{j}\;I_{i-j}\right)}$$

where $\rho_{j}$ corresponds to the discretized probability distribution of the generation interval and $I_{i}$ is the number of new cases on day *i* [25,29,63]. The generation interval is used along with past and current cases to determine how many cases contributed to the new cases. Dividing the new cases by this sum gives the average number of new cases that a single infected person subsequently caused and, therefore, the effective reproduction number $R_{t}$ [30]. For $I_{i}$, we can use the number of cases predicted by the model’s best fit, while the generation interval can be modeled using a discretized gamma distribution. In particular, we used a gamma distribution with a mean of 11 days and a variance of 5 day$s^{2}$ [64]. In [45], a mean of 6.5 days and a standard deviation of 2.9 days were assumed for the mean and variance for the infectious period of measles. In this case, they obtained a basic reproduction number between 12.5 and 18.

From Table 1, it can be seen that both effective reproduction numbers $R_{t}$ are greater than one and agree with previous post-vaccination estimations of the effective reproduction number $R_{t}$ [31]. In particular, in [65], the effective reproduction number $R_{t}$ was reported to be in the range of 1.6 to 4.7. The result in [65] was achieved assuming that 70% of the population at risk (children under 60 months) had immunity. Thus, our results are plausible despite all the spatio-temporal factors affecting the Texas–New Mexico measles 2025 outbreak. After computing the effective reproduction number $R_{t}$, we computed the basic reproduction number $R_{0}$ by using the following equation [62]:

(10) $$R_{0}=\frac{R_{t}}{1-pVE},$$

where pVE represents the fraction of people who are immune to measles. In particular, *p* is the proportion of people who received the measles vaccine and VE (dimensionless) is the efficacy of the measles vaccine. Based on the collected data and the scientific literature, we assume that the proportion of people who have received the measles vaccine is 95% and that the efficacy of the measles vaccine is 97% [6]. Thus, we obtain the basic reproduction number $R_{0}$ as 31.93 and 40.28, resulting from fitting the exponential growth model to new cases and cumulative cases, respectively. Note that we first need to compute the effective reproduction number $R_{t}$. In [66], a basic reproduction number $R_{0}$ of 22.1 and 32.1 was reported using data from Germany. Thus, again, our results are plausible despite the differences between Germany and the USA. We could also use the equation given in [25], where $R_{t}\approx R_{0}\;S\left(t\right)/N$, where *N* is the total population. In this case, we need an estimate of S(0) at the beginning of the outbreak. Note that we can use (1−pVE) as a proxy for S(0) and then obtain the same expression given in Equation (10).

In [62], the measles vaccine was estimated to be highly effective in preventing infection with an efficacy of 99.7%. Using this efficacy of the vaccine, we find that the basic reproduction number $R_{0}$ is 47.43 and 59.83 from the incidence and cumulative fits. These values are above those reported in other works but are still in agreement with a few works [31,54]. Thus, the efficacy of 99.7% is probably an overestimation. In fact, the effectiveness of the measles vaccine is normally considered to be 80–95% [67,68]. In [69], it was mentioned that the median efficacy of the vaccine with two doses is in the range of 94 to 100%. Then, if we assume that the efficacy of the vaccine is 94%, then the basic reproduction number $R_{0}$ is 23.42 and 29.55 from the incidence and cumulative fits. This also agrees well with various works [31]. We have used the Equation (8) to estimate the effective reproduction number $R_{t}$, but using Equation (9), we obtained results in similar ranges. In summary, we have shown that the results have uncertainty depending on several factors such as vaccine efficacy or vaccination coverage. However, the results are reasonable and agree with a few other works.

### 3.2. SIR Model Results

For the SIR model (5), we calibrate the model using the Texas–New Mexico measles 2025 outbreak cases. We chose to only use the first five weeks of the epidemic as after this point, the curve flattened and a similar number of new weekly cases were reported for multiple weeks. This behavior cannot be captured by the simple SIR model and would require the estimation of multiple transmission rates $\beta_{s}$ [70,71,72]. Since the model is still valid for this shorter time frame (window), we choose to use it to simplify our analysis. In [26], a significant analysis of the window and the relation to the growth rate is presented.

In this model, we estimate the transmission rate β, basic reproduction number $R_{0}$, and the effective reproduction number $R_{t}$. In particular, in the SIR model, we use $R_{t}\approx R_{0}\;S\left(t\right)$. This approach is easier than using the generation interval Equation (9), which requires several assumptions and more parameters. For instance, the size of the time window for the fits plays a role on the estimation of the effective reproduction number $R_{t}$ [14,26]. For the SIR model, we need initial conditions for the three subpopulations or classes. Since there are people immune to measles, we need to remove those from the susceptible population [31,62]. People who are not susceptible to measles (mainly vaccinated people) can be assigned to the recovered population R(t). To determine the size of the total population (N) and recovered and susceptible subpopulations, we used the US Census Bureau’s county level population data (open access from website) [73]. We looked at five counties, Lea, Dawson, Gaines, Terry, and Yoakum, as these counties have a higher number of cases of measles. Thus, we estimated the total population of the region where the Texas–New Mexico measles outbreak was concentrated. We then assigned people to the two subpopulations using an estimated vaccination coverage of 0.95 [6]. Thus, R(0)=0.95N and S(0)=0.05N. For the initial infected population, we used the number of cases reported during the first week of the outbreak.

**Figure 3:**
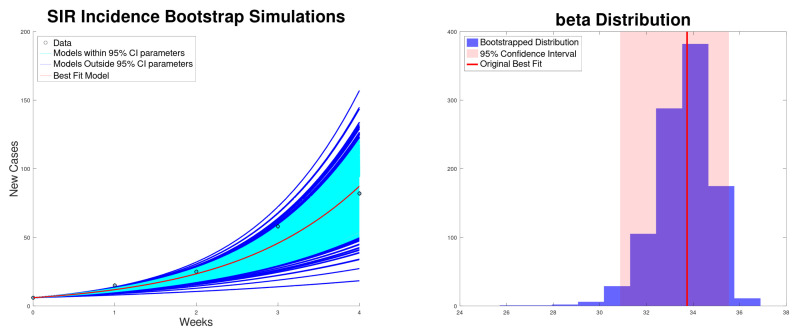
Best fit to the new cases, along with the 95% confidence interval (bootstrapping) of the SIR model (5) to the Texas–New Mexico measles 2025 outbreak (**left**). Bootstrapping distribution of the transmission rate β with the 95% confidence interval (**right**).

**Figure 4:**
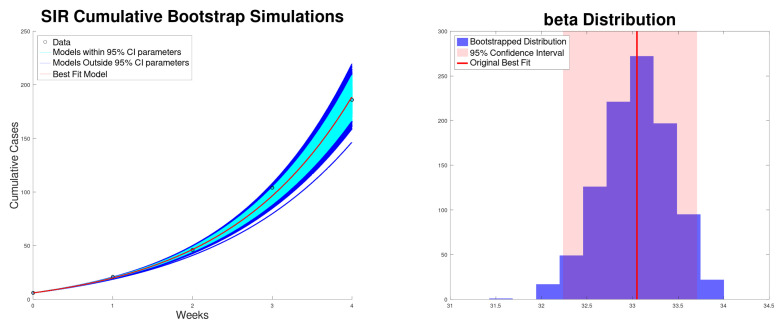
Best fit of the cumulative cases, along with the 95% confidence interval (bootstrapping) of the SIR model (5) to the Texas–New Mexico measles 2025 outbreak (**left**). Bootstrapping distribution of the transmission rate β with the 95% confidence interval (**right**).

Figure 3 shows the best fit of the SIR model (5) to the new cases of the Texas–New Mexico measles 2025 outbreak. The model fits well with the measles data. Figure 4 shows the best fit of the SIR model (5) to the cumulative cases of the Texas–New Mexico measles 2025 outbreak only during the increasing phase. Again, the SIR model fits well with the measles data.

Table 2 shows the results of the calibration of the SIR model (5) for the Texas–New Mexico measles 2025 outbreak cases. It can be seen that the effective reproduction number $R_{t}$ is greater than one and is in agreement with previous post-vaccination estimations of the effective reproduction number $R_{t}$ [31]. Moreover, these results are in accordance with the scientific literature on measles data in different regions around the world [31,62,65,66]. The ranges presented in Table 2 for the effective reproduction number $R_{t}$ are calculated using $R_{t}=R_{0}\;S\left(0\right)/N$ and $R_{0}=\beta /\gamma$.

When using new cases per week, we estimated the parameters by using MATLAB’s fmincon built-in function to minimize the following objective function:

(11) $$\min_{\beta_{i}}SSR=\min_{\beta_{i}}\sum_{j}{\left(I_{j}-I_{j}\right)}^{2},$$

where $I_{j}$ is the number of reported measles cases that occurred in week jth. We used an infectious period of 7 days, which allows us to use new cases per week. The scientific literature related to measles shows variability with regard to the infectious period, but our assumption is in the range of other studies [31,33,45,74,75]. Thus, the only parameter that is estimated is the transmission rate [15]. A similar approach is used when we use cumulative cases, but the objective function includes cumulative cases. In this case, we need an extra equation in the SIR model for computing the cumulative cases. This equation tracks the total number of people who at some point become infected.

### 3.3. SEIR Model Results

For the mechanistic SEIR model (6), we calibrate the model using new cases per week and cumulative cases of the Texas–New Mexico measles 2025 outbreak. We examine the same growth phase as in the SIR model and use the same initial conditions of 6446 and 122,478 for the susceptible and recovered subpopulations. These approximations are made using the following values: R(0)=0.95N and S(0)=0.05N. For the initial infected population, we use the number of reported cases during the first week of the outbreak. Performing the calibration of the SEIR model, we estimate the transmission rate β, basic reproduction number $R_{0}$, and the effective reproduction number $R_{t}$.

**Figure 5:**
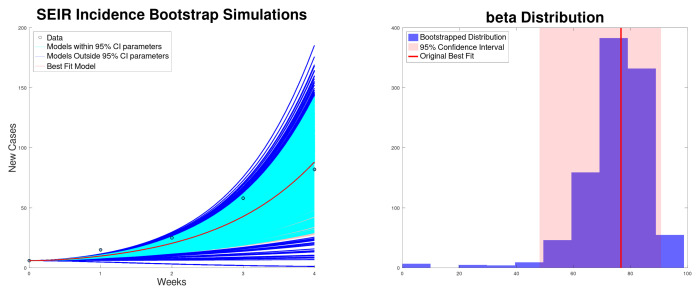
Best fit of the new cases, along with the 95% confidence interval (bootstrapping) of the SEIR model (5) to the Texas–New Mexico measles 2025 outbreak (**left**). Bootstrapping distribution of the transmission rate β with the 95% confidence interval (**right**).

**Figure 6:**
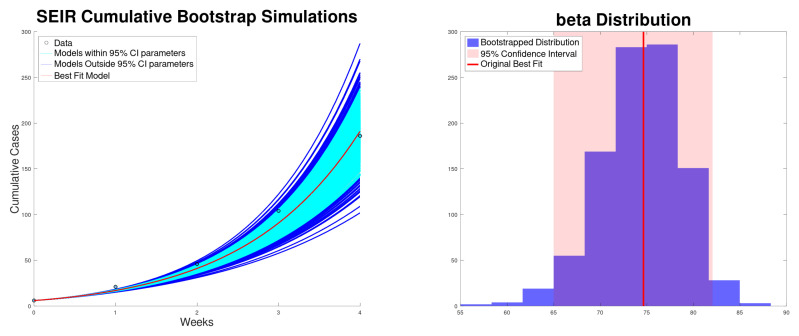
Best fit of the cumulative cases, along with the 95% confidence interval (bootstrapping) of the SEIR model (5) to the Texas–New Mexico measles 2025 outbreak (**left**). Bootstrapping distribution of the transmission rate β with the 95% confidence interval (**right**).

Figure 5 shows the best fit of the SEIR model (6) to the new cases from the Texas–New Mexico measles 2025 outbreak. The model fits well with the measles data. Figure 6 shows the best fit of the SEIR model (6) to the cumulative cases of the Texas–New Mexico measles 2025 outbreak. The model fits well with the measles data.

Table 2 shows the results of the calibration of the SEIR model (6) for the Texas–New Mexico measles 2025 outbreak cases. It can be seen that the effective reproduction number $R_{t}$ is greater than one and is in good agreement with previous post-vaccination estimations of the effective reproduction number $R_{t}$ [31]. Moreover, these results are in accordance with the scientific literature on measles data in different regions around the world [31,62,65,66]. Again, we estimated the parameters by using the MATLAB’s fmincon built-in function to minimize the objective function in Equation (11) when new cases per week are used. A similar approach is used when the cumulative cases are fitted. We used an infectious period of 7 days and a latent period of 11 days [38]. Therefore, the only parameter that needs to be estimated is the transmission rate β [15].

### 3.4. Sensitivity Analysis of SIR and SEIR Model’s Results

In this section, we perform additional computations with regard to fits of the SIR and SEIR mathematical models to observe the changes in the results with different infectious and latent periods. This is a sensitivity analysis and uncertainty quantification.

Table 3 shows the estimates of the effective reproduction number $R_{t}$ and $R_{0}$ using the best fit of the mechanistic SEIR model (6) to the Texas–New Mexico measles 2025 outbreak cases, with different infectious periods. In addition, it assumes different percentages of vaccinated people. It can be observed that if the infectious period is shorter than the assumed value of 7 days, $R_{t}$ and $R_{0}$ decrease, but this is in the range of previous results (still above average of the previous outbreaks). When the percentage of vaccinated people is lower, $R_{t}$ and $R_{0}$ decrease.

Table 4 shows the estimates of the effective reproduction number $R_{t}$ and $R_{0}$ using the best fit of the mechanistic SEIR model 6 to the Texas–New Mexico measles 2025 outbreak cases, with different infectious and latent periods. In addition, assuming different percentages of vaccinated people and initial exposed individuals. It can be observed that if the infectious period is shorter than the assumed value of 7 days, one understands that $R_{t}$ and $R_{0}$ decrease, but in the range of previous results (still above average of previous outbreaks). With regard to the latent period when it is shorter than the assumed value of 11 days, one understands that $R_{t}$ and $R_{0}$ decrease, but still above the average of previous outbreaks. When the percentage of vaccinated people is lower, one obtains that $R_{t}$ and $R_{0}$ decrease. Similarly, this occurs when the percentage of the initial exposed subpopulation is higher than the original assumed value. This is likely to be true since, in the initial growth phase of the outbreak, cases are increasing. In summary, the sensitivity analysis shows that potential lower and higher values of $R_{t}$ and $R_{0}$ could be possible.

## 4. Discussion

The results presented in this work are consistent with the scientific literature related to the basic reproduction number of measles disease. However, the results of previous studies on the basic reproduction number of measles are very broad [31,54]. Obviously, as in any mathematical modeling study, all these results are subject to assumptions regarding the data and models. For instance, to compute the effective reproduction number using the mechanistic SIR and SEIR models, assumptions about the percentage of the population immune to measles are needed. Therefore, the efficacy of the measles vaccine and the proportion of people vaccinated are also needed. In addition, previous studies have worked with a variety of hypotheses. In this paper, we used a gamma distribution to compute the effective reproduction number for the exponential growth model. For the mechanistic SIR and SEIR models, we used the classical approximation that depends on the susceptible population to compute the effective reproduction number.

An interesting discussion is about which model is more suitable to assess the growth of an epidemic [14,17,26,46]. Some researchers have argued that mechanistic models for estimating the growth rate or basic reproduction umber $R_{0}$ should not rely on detailed knowledge of the disease transmission process, as it is sometimes unclear in the early phase of an outbreak of an emerging disease [14]. However, we believe that for measles outbreaks, there is historical information available such that the use of a SIR or SEIR model seems appropriate to estimate the growth of the outbreak.

For the exponential growth model, we computed and estimated that the growth rate is approximately 0.66 and 0.86, using incidence and cumulative cases, respectively. The estimated basic reproduction number was approximately 32 and 40 when fitting the model to new cases and cumulative cases. These results are in good agreement with previous results from the post-vaccine era [31]. In particular, in the measles outbreak in the region of North Rhine-Westphalia, Germany, a basic reproduction number between 22.1 and 32.1 was estimated [62]. In other studies from the pre- and post-vaccine era in countries such as Niger, Senegal, Kenya, Tanzania, Zaire, Uganda, Cameroon, Zambia, and India, a basic reproduction number between 3.7 and 203.3 was estimated [75].

For the classical SIR model, we estimated that the basic reproduction number is approximately 30 regardless of the use of incidence and cumulative cases. This, as before, is in good agreement with previous post-vaccine era results [31,62,75]. With regard to the SEIR model, we obtained a basic reproduction number of roughly 75 regardless of the use of incidence and cumulative cases. In this case, we have values higher than some of the values reported in previous works for the post-vaccine era [31,62,75]. There are several potential explanations for this higher basic reproduction number. For example, we performed a sensitivity analysis of the SIR and SEIR models with regard to the percentage of people immune to measles in the counties of Texas and New Mexico. Considering that 98% of people are immune, we find that the basic reproduction number in the SIR model and using cumulative cases becomes approximately 83. On the other hand, if we consider that 92% of the people were immune, then we understand that the basic reproduction number is approximately 20, which is closer to other works. Thus, we think that it might be possible that the reported vaccinated people are higher than the real one, and then lower values of the basic reproduction number could be possible. In addition, using an infectious phase of 5 days decreases the basic reproduction number to approximately 28. With regard to the results of the SEIR model, we obtained a larger basic reproduction number. There are several potential explanations for this. For example, considering a latent phase of 7 days instead of 11 days reduces the basic reproduction number to approximately 59. Recently, incubation periods have been reported as low as 7 days for infectees without a vaccination history [76]. In [39], an incubation period of 3.5 days was also used and this significantly reduces the basic reproduction number. In addition, in the sensitivity analysis of the SEIR model, we considered that 92% of the people are immune, which decreases the estimate of the basic reproduction number, which is closer to other works. Thus, we think that it might be possible that the reported vaccinated people are higher than the real one. Another aspect that reduces the basic reproduction number in the SEIR model is to increase the initial number of exposed individuals. In our study, we assumed that the initial number of exposed individuals is proportional to the incubation period and the initial infected cases. However, in the sensitivity analysis, we increased the initial exposed individuals by 50% and the basic reproduction number was reduced to approximately 68. Since the model was fitted to the initial growth phase, it is reasonable to assume that there are more initial exposed individuals than the initial number of infectees, even when assuming the incubation period to be equal to the infectious phase. Thus, due to several uncertainties, related to the SEIR model, the basic reproduction number could be lower. It is important to note that the SEIR model provided larger values for $R_{0}$ than the SIR model, but this difference can be reduced as mentioned before by increasing the initial number of exposed individuals or reducing the latent period. These two factors have uncertainty and therefore the difference on the estimates of $R_{0}$ can be smaller. Additionally, considering that 92% of the people are immune, we obtain from the SEIR model that the basic reproduction number becomes approximately 46, which is closer to other works. Thus, the larger estimations for the basic reproduction number might be due to lower vaccination coverages than the reported ones. However, assuming that the reported vaccinated people are accurate, then the results imply that the basic reproduction number of the Texas–New Mexico measles outbreak is higher than other measles outbreaks [31]. This could be due to people having more contact with other people during the outbreak compared to other outbreaks. These two previous factors have been mentioned as riskier factors for the Texas–New Mexico measles outbreak [77,78].

Previous works have proposed a variety of models to study measles dynamics using different points of view [25,32,40]. Some works have used the SIR and SEIR models [33,42]. In [33], it is mentioned that for the SIR model, the infectious period can be assumed as 13 days, whereas for the SEIR model, the exposed stage lasts 8 days with an infectious period of 5 days. In [45], an effective reproduction number between 12.5 and 18 was reported. In [31], a systematic review of works that have estimated the basic reproduction number $R_{0}$ of measles is presented. The review covered works that made estimations pre-vaccine era and post-vaccine era. Thus, estimates of the effective reproduction number $R_{t}$ are also presented. With regard to the former, a basic reproduction number $R_{0}$ for measles has been reported to be up to 770 and a minimum value of 1.43. For the post-vaccine era, a maximum value of 32.1 and a minimum value of 4.6 were reported. All these results show that the usual range of 12–18 is not always reliable. Thus, in [31], the authors highlight the importance of estimating the basic reproduction number for different regions around the world using local data. In this way, we can provide more robust data and help to understand the dynamics of measles.

As in any mathematical modeling study, there are limitations. One that is very commonin epidemiology is related to unreported cases that are crucial in many epidemics and are difficult to quantify. For instance, if there are unreported cases, the results presented in this work are underestimating the epidemic growth rate. However, if we take into account that the percentage of vaccinated people is lower than we estimated, then the growth rate could be smaller. Thus, there is a trade-off in the results depending on the unreported cases and the level of vaccinated people against measles. Nevertheless, we think that the number of unreported cases can be more determinant of the growth rate since the level of vaccination coverage can be more reliable [9,79]. As in other studies, we cannot draw strong conclusions due to some uncertainty in some parameters of the model. In [43], a few contradictory observations regarding the quality of the fit were obtained and the basic reproduction numbers were estimated, providing a wide range.

Finally, the results presented in this work provide useful insight into measles outbreaks in the post-vaccine era [31]. In particular, we provided a characterization of the Texas–New Mexico 2025 measles outbreak. The results with respect to the growth rate, the basic reproduction number, and the effective reproduction number show that the outbreak has higher values than other previous measles outbreaks but which are still lower than others [31,75]. We provided a discussion of the potential reasons behind these differences. John Hopkins data on MMR vaccinations indicate that other counties have low vaccination rates similar to some of the affected Texas counties, and therefore measles outbreaks have the potential to appear in the future [80]. The estimates provided in this work can help health institutions implement interventions to reduce the effective reproduction number and achieve herd immunity. For example, previous studies have mentioned and estimated the required percentage of vaccinated people to eradicate measles [81,82,83]. Thus, interventions such as vaccination can reduce the effective reproduction number.

Future research directions can include similar studies to estimate the growth of other measles outbreaks and the use of alternative statistical techniques, such as Bayesian methods, to enhance uncertainty quantification. This would allow better understanding of the spread of measles in the population and avert deaths. Another future aspect that can be investigated is the underreported cases, although due to the severity of measles disease, it is less likely than in influenza or COVID-19 [84]. If the number of underreported cases is large, then the effective and the basic reproduction number might be higher. In 2025, 27 outbreaks have been reported in the USA and 1130 confirmed cases have been associated with these outbreaks [6]. Thus, research on this topic is very relevant for public health.

## Figures and Tables

**Table 1:** Growth rate *r* (da$y^{1}$) estimated from the best fit of the exponential growth model (2) to the Texas–New Mexico measles 2025 outbreak cases. Estimation of the effective reproduction number $R_{t}$ and $R_{0}$ using Equations (8) and (10). In addition, the 95% confidence intervals are provided.

	Growth Rate *r* (95% CI)	$R_{t}$ (95% CI)	$R_{0}$ (95% CI)	SSR
Incidence	0.669 (0.536–0.748)	2.51 (2.12–2.75)	31.93 (27.08–35.06)	220
Cumulative	0.869 (0.765–0.936)	3.16 (2.80–3.41)	40.28 (35.74–43.39)	762

**Table 2:** Estimates of the effective reproduction number $R_{t}$ and $R_{0}$ using the best fit of the mechanistic SIR and SEIR models to the Texas–New Mexico measles 2025 outbreak cases. In addition, the 95% confidence intervals.

Model	β (da$y^{-1}$) (95% CI)	Range of $R_{t}$ (95% CI)	$R_{0}$ (95% CI)	SSR
SIR incidence	33.7 (30.9–35.5)	1.69 (1.54–1.77)	33.7 (30.9–35.5)	192
SIR cumulative	33.1 (32.4–33.7)	1.65 (1.61–1.68)	33.1 (32.4–33.7)	67
SEIR incidence	76.6 (48.1–90.7)	3.82 (2.40–4.52)	76.6 (48.1–90.7)	318
SEIR cumulative	74.6 (65.0–82.1)	3.72 (3.24–4.09)	74.6 (65.0–82.1)	243

**Table 3:** Sensitivity analysis of the SIR model. Estimates of the effective reproduction number $R_{t}$ and $R_{0}$ using the best fit of the SIR model (5) to the Texas–New Mexico measles 2025 outbreak cases.

SIR Sensitivity	Incidence		Cumulative		
	$R_{0}$	$R_{t}$	$R_{0}$	$R_{t}$	
Original	33.7	1.68	33.0	1.65	
92% Vaccinated	21.0 ▾	1.68	20.59 ▾	1.65	← lower $R_{0}$
98% Vaccinated	85.7 ▴	1.71	83.6 ▴	1.67	← higher $R_{0}$
5 day infectious period	N/A		28.34 ▾	1.42	
8.7 day infectious period	N/A		37.14 ▴	1.85	

**Table 4:** Sensitivity analysis of the SEIR model. Estimates of the effective reproduction number $R_{t}$ and $R_{0}$ using the best fit of the SEIR model (6) to the Texas–New Mexico measles 2025 outbreak cases.

SEIR Sensitivity	Incidence		Cumulative		
	$R_{0}$	$R_{t}$	$R_{0}$	$R_{t}$	
Original	76.6	3.82	74.6	3.72	
92% Vaccinated	47.6 ▾	3.80	46.4 ▾	3.71	← lower $R_{0}$
98% Vaccinated	197.3 ▴	3.92	190.9 ▴	3.80	← higher $R_{0}$
5 day infectious period	N/A		63.5 ▾	3.17	← lower $R_{0}$
8.7 day infectious period	N/A		84.3 ▴	4.20	
7 day latent period	60.8 ▾	3.04	59.3 ▾	2.96	← lower $R_{0}$
15 day latent period	92.6 ▴	4.62	90.1 ▴	4.49	← higher $R_{0}$
50% higher E(0)	71.4 ▾	3.56	68.3 ▾	3.41	

## Data Availability

The original contributions presented in this study are included in the article. Collected data are available from public websites and links provided in the references. Further inquiries can be directed to the corresponding author.
